# Paracoccidiodomicosis suprarrenal

**DOI:** 10.7705/biomedica.4844

**Published:** 2020-08-20

**Authors:** Alejandro Román-González, Juan Pablo Toro, Luis F. Arias

**Affiliations:** 1 Departamento de Medicina Interna, Hospital Universitario San Vicente Fundación, Universidad de Antioquia, Medellín, Colombia Universidad de Antioquia Departamento de Medicina Interna Universidad de Antioquia Medellín Colombia; 2 Departamento de Cirugía, Hospital Universitario San Vicente Fundación, Universidad de Antioquia, Medellín, Colombia Universidad de Antioquia Departamento de Cirugía Universidad de Antioquia Medellín Colombia; 3 Departamento de Patología, Universidad de Antioquia, Medellín, Colombia Universidad de Antioquia Departamento de Patología Universidad de Antioquia Medellín Colombia

**Keywords:** paracoccidioidomicosis, glándulas suprarrenales, hidrocortisona, prednisona, prednisolona, Paracoccidioidomycosis, adrenal glands, hydrocortisone, prednisone, prednisolone

## Abstract

La insuficiencia suprarrenal primaria es un defecto en la producción de glucocorticoides, mineralocorticoides y andrógenos sexuales. Los pacientes afectados por esta condición se caracterizan por concentraciones bajas de cortisol y deficiencia de aldosterona con hiponatremia e hiperpotasemia concomitantes.

La etiología más común es el desarrollo de anticuerpos contra la enzima 21 hidroxilasa. Otra causa importante de la insuficiencia suprarrenal primaria son las enfermedades infecciosas, en especial en los países de bajos ingresos. Entre las causas infecciosas que se han descrito se encuentran: *Mycobacterium tuberculosis*, el complejo de *Mycobacterium avium*, *Neisseria meningitidis*, *Pseudomonas aeruginosa, Haemophilus influenzae,* citomegalovirus*, Pneumocystis jirovecii, Histoplasma capsulat*um, *Blastomyces dermatiditis*, *Cryptococcus neoformans*, *Cocciodiodes immitis*, *Nocardia* spp. y *Paracoccidioides brasiliensis*.

En este artículo se presenta la imagen de la tomografía de un paciente que presentó falla suprarrenal, con masas en las glándulas suprarrenales, cuya biopsia permitió establecer el diagnóstico final de paracoccidioidomicosis.

## Abstract (en)

Primary adrenal insufficiency is a defect in glucocorticoid, mineralocorticoid and sexual androgens production. Patients with this disorder have low cortisol levels and aldosterone deficiency with concomitant hyponatremia and hyperkalemia.

The most common etiology of this disease is the production of antibodies against the enzyme 21 hydroxylase. Another common cause, particularly in low income countries, are infectious diseases. Several micro-organisms have been reported as a causal agent in adrenal insufficiency including *Mycobacterium tuberculosis*, *Mycobacterium avium comple*x, *Neisseria meningitidis*, *Pseudomonas aeruginosa*, *Haemophilus influenzae*, cytomegalovirus, *Pneumocystis jirovecii*, *Histoplasma capsulatum*, *Blastomyces dermatiditis*, *Cryptococcus neoformans*, *Cocciodiodes immitis*, *Nocardia* spp. and *Paracoccidioides brasiliensis*.

In this article, we present the computerized tomography and the adrenal biopsy of a patient with adrenal insufficiency. The final diagnosis was paracoccidioidomycosis.

La insuficiencia suprarrenal primaria es una enfermedad infrecuente con una prevalencia estimada de 100 a 140 casos por millón de habitantes ^1^. La causa principal en los países desarrollados es la autoinmunidad contra la glándula suprarrenal. En los países de escasos o medianos recursos, o en las economías de transición, la causa más común son las enfermedades infecciosas, siendo la tuberculosis la causa principal. Se han descrito otras causas infecciosas como *Mycobacterium avium* complex, meningococo, *Pseudomonas aeruginosa*, *Haemophilus influenza*, citomegalovirus, *Pneumocystis jiroveci*, *Histoplasma capsulatum*, *Blastomyces dermatiditis*, *Cryptococcus neoformans*, *Cocciodiodes immitis* y *Nocardia* spp. Una causa infecciosa rara es la paracoccidioidomicosis, una micosis sistémica endémica en Colombia, Venezuela y Brasil ^2^.

En los pacientes con insuficiencia suprarrenal primaria y con anticuerpos negativos contra la 21-hidroxilasa, se requiere una imagen tomográfica de las glándulas suprarrenales ^2^. Su engrosamiento sugiere una condición infecciosa, infiltrante o maligna. Por ende, cuando la historia clínica y los exámenes físicos y paraclínicos no indican la causa subyacente, puede requerirse una biopsia para determinar el origen de la insuficiencia suprarrenal. En este sentido, en los países donde las enfermedades infecciosas son una causa frecuente de falla suprarrenal, la biopsia de esta glándula puede ser útil.

En este artículo, se presenta la imagen de la tomografía de un paciente que presentó falla suprarrenal, con masas en las glándulas suprarrenales, cuya biopsia confirmó el diagnóstico final de paracoccidioidomicosis.

## Caso clínico

Se trata de un hombre de 62 años de edad con antecedentes de enfermedad pulmonar obstructiva crónica, que colsultó al servicio de urgencias por un cuadro clínico de seis meses de evolución con pérdida de peso, fatiga, náuseas, dolor abdominal, diaforesis y fiebre.

En el examen físico se encontró hipotensión arterial (84/45 mm Hg), taquicardia (110 latidos por minuto) e hiperpigmentación generalizada. En los exámenes de laboratorio se reportó hiponatremia e hiperpotasemia, con disminución del cortisol (0,74 µg/dl; rango de referencia: 6 a 23 µg/dl; menor de 5 µg/dl: sugestivo de insuficiencia suprarrenal) y aumento de la corticotropina (ACTH) (mayor de 1.250 pg/ml; rango de referencia: 6 a 76 pg/ml).

**Figura 1:**
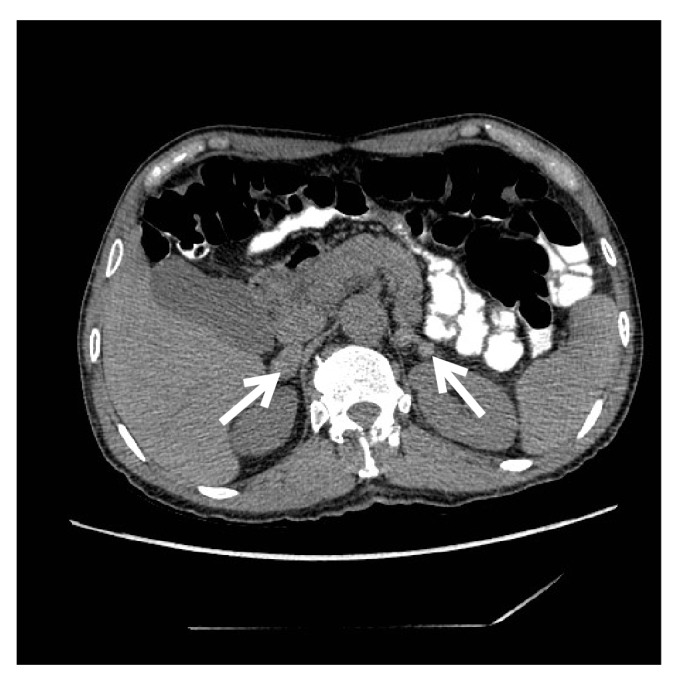
Masas en las glándulas suprarrenales (flechas)

Con base en estos datos, se diagnosticó una insuficiencia suprarrenal primaria. En la tomografía abdominal con contraste, se observaron masas en las glándulas suprarrenales, la derecha de 26 x 14 mm y la izquierda de 33 x 17 mm (figura 1), con un bajo lavado del contraste. En la tomografía de tórax, se observó enfisema.

**Figura 2:**
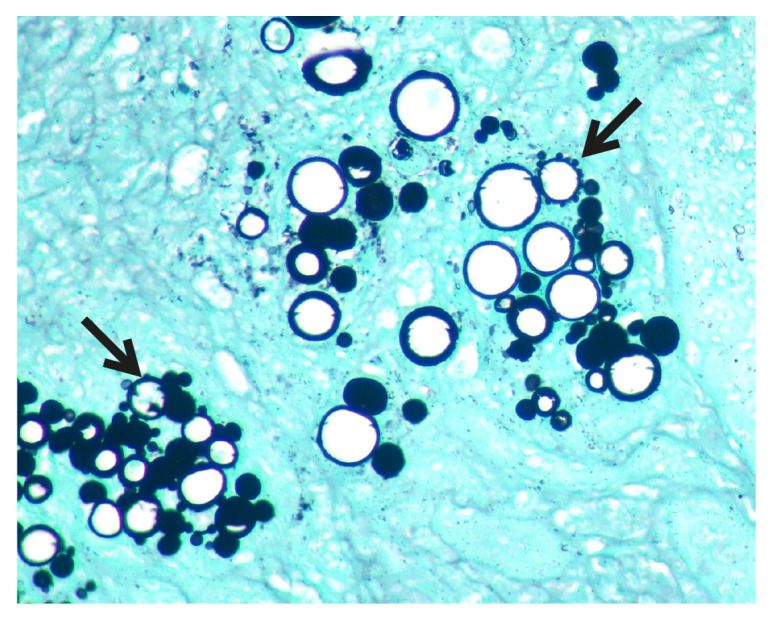
*Paracoccidioides brasiliensis* detectado en la biopsia de la masa de la glándula suprarrenal. Se aprecian las imágenes típicas del hongo y los brotes radiales (‘rueda de timón’) en el cuadrante superior derecho y en el inferior izquierdo (flechas). Plata metenamina, 400X

Mediante lavado broncoalveolar se descartaron procesos infecciosos pulmonares, como la tuberculosis. Por laparoscopia, se tomó una biopsia de una de las masas suprarrenales, de la cual se aisló *Paracoccidioides brasiliensis* (figura 2, plata metenamina).

El paciente fue tratado con 5 mg diarios de prednisolona, 0,1 mg diarios de fluorocortisona y 200 mg de itraconazol cada 8 horas, durante seis meses. Dos años después del diagnóstico, el paciente se encuentra vivo, asintomático y en tratamiento para su insuficiencia suprarrenal.

### Consideraciones éticas

Se siguieron las normas éticas para la investigación en seres humanos contenidas en la Resolución 008430 de 1993 del Ministerio de Salud de Colombia y se mantuvo la confidencialidad del paciente, pues sus datos se manejaron en forma anónima.

## References

[1] Bornstein SR, Allolio B, Arlt W, Barthel A, Don-Wauchope A, Hammer GD (2016). Diagnosis and treatment of primary adrenal insufficiency: An endocrine society clinical practice guideline. J Clin Endocrinol Metab.

[2] Colombo AL, Tobón A, Restrepo A, Queiroz-Telles F, Nucci M (2011). Epidemiology of endemic systemic fungal infections in Latin America. Med Mycol.

